# FAT10 Silencing Prevents Liver Fibrosis through Regulating SIRT1 Expression in Hepatic Stellate Cells

**DOI:** 10.7150/ijms.77367

**Published:** 2023-02-27

**Authors:** Weihong Zheng, Fei Guan, Guoxing Xu, Yikai Yu, Jun Xiao, Xiaowei Huang

**Affiliations:** 1 Department of General Surgery, Tongji Hospital, Tongji Medical College, Huazhong University of Science and Technology, Wuhan 430030, China; 2 Department of Pathogen Biology, School of Basic Medicine, Tongji Medical College, Huazhong University of Science and Technology, Wuhan 430030, China; 3 Department of Respiratory and Critical Care Medicine, The Central Hospital of Wuhan, Tongji Medical College, Huazhong University of Science and Technology, Wuhan 430010, China; 4 Department of Rheumatology, Tongji Hospital, Tongji Medical College, Huazhong University of Science and Technology, Wuhan 430030, China; 5 Department of Gastroenterology and Hepatology, Huangzhou District People's Hospital, Huanggang 438000, China; 6 Department of Gastroenterology and Hepatology, Tongji Hospital, Tongji Medical College, Huazhong University of Science and Technology, Wuhan 430030, China

**Keywords:** FAT10, hepatic stellate cells, SIRT1, liver fibrosis, inflammation

## Abstract

**Background and objectives:** Hepatic stellate cell (HSC) activation is the cardinal factor due to the accumulation of extracellular matrix proteins during the development of liver fibrosis. The aim of the present study was to find new targets for developing drugs to treat liver fibrosis, by screening the key genes involved in the activation of hepatic stellate cells.

**Methods:** Differentially expressed genes were identified through TCGA database. RT-PCR, immunohistochemistry (IHC) assay, western blot, and ELISA were performed to evaluate the expression levels of FAT10 and fibrotic molecules. *In vitro* experiments were conducted to investigate the signaling pathways and biological functions of FAT10 in LX-2 cell lines.

**Results:** In the present study, expression profiles obtained from the Gene Expression Omnibus (GEO) were used to explore the different genes expression between HSCs treated with or without carbon tetrachloride (CCl4). Human leukocyte antigen (HLA)-F adjacent transcript 10 (FAT10) was selected for further investigations. In animal model of carbon tetrachloride-induced liver fibrosis, the expression of FAT10 on activated HSCs is upregulated. *In vitro*, silencing FAT10 reduced TGF-β1-induced ECM activation and accumulation in LX-2 cells, and also suppressed the inflammatory response of LX-2 cells. Further Transwell results suggested that knockdown of FAT10 could inhibit TGF-β1-induced LX-2 cell migration and invasion. Mechanistically, FAT10 promotes its fibrotic activity through regulating sirtuin 1 (SIRT1), with a concomitant activation of ECM.

**Conclusions:** These findings indicated an unexpected role of FAT10 in liver fibrosis development, suggesting that silencing FAT10 might represent a new strategy for the treatment of fibrotic liver diseases.

## Introduction

Liver fibrosis is a multiple process disease and the main pathological repair response to chronic injuries, characterized by the excessive deposition and abnormal distribution of extracellular matrix (ECM) and persistent inflammatory response 1-4. The imbalance between the production and degradation of ECM led to the development of cirrhosis and liver failure, which resulted in a worldwide health problem 5-7. A great number of evidences have shown that activation of hepatic stellate cells (HSCs), a minor cell population in normal liver, played a key role in hepatic fibrosis 8, 9. However, no effective treatment has been approved to completely reverse liver fibrosis in clinical application 5, 10, 11. Herein, it is urgent to explore an effective anti-hepatic fibrosis method.

HSCs were mainly located in cavity of Disse surrounding the hepatic sinusoids 12. HSCs were activated by various pathogenic factors of liver injury, then transformed to myofibroblast-like cells 4, 13, 14. Myofibroblast-like cells expressed α-SMA and secreted pro-inflammatory factors and extracellular matrix components 15-17. FAT10, a member of ubiquitin-like modifier family, was reported to be involved in multiple biological functions, including protein translocation, cell-mediated immunity, signal transduction, and cell cycle regulation through binding to its target proteins and subjecting them to degradation 18-20. Several studies have shown that FAT10 has a crucial role in hepatocellular carcinoma (HCC) development by a variety of biological behaviors 21-23. In addition, a recent study suggested that FAT10 was not only increased in patients with HCC, but also *in vivo* experiments model 24. However, the role of FAT10 in liver fibrosis remains largely unknown.

In this study, by use of the Gene Expression Omnibus (GEO) database, FAT10 was found to be significantly upregulated in fibrotic liver tissues, as well as in activated HSCs. *In vitro* experiments were performed to explore the function of FAT10 in ECM accumulation and inflammatory response of HSCs. Furthermore, our data showed that FAT10 promoted ECM accumulation and inflammatory response through modulating SIRT1 degradation.

## Materials and Methods

### Bioinformatics Analysis

The RNA sequencing datasets GSE149508 8 and GSE167216 25 were downloaded from the Gene Expression Omnibus (GEO) database. GSE149508 contains the gene expression data between quiescent and activated hepatic stellate cells. GSE167216 contains the gene expression data among normal liver tissues, liver fibrosis models with 6 or 12-months damage induction by CCl4. The data were analyzed using easyGEO (https://tau.cmmt.ubc.ca/eVITTA/easyGEO) 26. The differentially expressed genes (DEGs) was selected with |log2 multiple change (FC)| >1.5 and *P*-value <0.01.

### Cell culture and treatment

The immortalized human hepatic stellate cell line LX-2 was a gift from Professor Lieming Xu (Shanghai Univesity of TCM, Institute of Liver Diseases, Shuguang Hospital, Shanghai, China) 27 and was detected to be free of mycoplasma. Cells were cultured in Dulbecco's modified Eagle's medium (HyClone, Logan, UT, USA) supplemented with 10% fetal bovine serum (Life Technologies, Grand Island, NY, USA) at 37°C in a humidified environment containing 5% CO2. A recombinant human TGF-β1 (Protein Tech Group, Inc, Chicago, USA) at a concentration of 10 ng/mL was added to the cell culture after FAT10 knockdown for detection of fibrotic factors and inflammatory cytokine secretion.

### A mouse model of liver cirrhosis

C57BL/6J mice (male, 4-5 weeks) were obtained from Shanghai ZY Laboratory Animal Co., Ltd (Shanghai, China). All animal experiments were conducted in accordance with its Animal Experiment Guidelines and approved by Ethics Committee of Tongji Hospital of Tongji Medical College, Huazhong University of Science and Technology. To induce liver fibrosis, C57BL/6J mice (n= 8) received corn oil (Control) or CCl4 (dissolved in corn oil, 0.1 ml/100 g body weight) by intraperitoneal injection, twice a week for 24 weeks. Liver samples were collected for further staining.

### Quantitative Real-Time PCR

Total RNA was extracted from tissues and cells using Trizol reagent (Life Technologies) according to the manufacturer's instructions. Complementary DNA (cDNA) was synthetized using PrimeScript™ RT reagent Kit (Takara Biomedical Technology Co., Ltd., Beijing, China). qRT-PCR was conducted using SYBR Green PCR Master Mix (Applied Biosystems, USA) on a 7500HT Fast Real-Time PCR System (Applied Biosystems) with GAPDH chosen to be the reference gene. The primers were synthesized by Sangon (Shanghai, China) and were listed in Table 1.

### Cell transfection and stable cell lines

pLKO.1-TRC cloning vector mediated short hairpin RNA (shRNA) targeting FAT10 was generated according to the manufacturer's protocol. Transfection was performed using Lipofectamine 3000 (Life Technologies) according to the manufacturer's protocol. The sequence of the FAT10-targeting short hairpin RNA (shRNA) was as follows: shFAT10#1, 5'-GGAATGGGATTTAATGACCTT-3'; shFAT10#2, 5'-GGGAAGATGATGGCAGATT-3'; SCR, 5'-ACGCATGCATGCTTGCTTT-3'. LX-2 cells were infected by the lentivirus and selected with 2 μg/mL puromycin (Sigma-Aldrich, Milwaukee, WI, USA) for about two weeks to obtain stably transfected cell lines.

### Enzyme-Linked Immunosorbent Assay (ELISA)

The cell supernatant was collected and the levels of IL-1β, IL-6, and TNF-α were determined by ELISA according to the manufacture's protocols (Sigma-Aldrich).

### Transwell assays

The cellular migration and invasion ability was measured using chambers with transwell inserts of 8 µm in pore size (Corning Inc., Corning, USA) without or with Matrigel (BD Biosciences, NJ, USA). About 8×10^4^ cells in 150 µL serum free medium were seeded in the upper chamber, complete medium supplemented with 20 ng/ml VEGF was placed in the lower chamber. Cells were fixed with 4% paraformaldehyde and stained with 1% crystal violet after 24 h seeding. The migration or invasion cells were captured in 6 random visual fields using a microscope (Olympus Corporation).

### Western Blot analysis

Proteins were extracted from cells on ice with RIPA lysis buffer (Cell Signaling Technology, Beverly, MA, USA) containing protease inhibitor cocktail (Sigma-Aldrich). The subsequent Western blotting analysis was performed using standard procedure. The primary antibodies used were listed as follows: FAT10 (Merck Millipore, Billerica, MA, USA); SIRT1 (Cell Signaling Technology); α-SMA (Protein Tech, China); fibronectin (ProteinTech Group, Inc.); COL1A1 (Cell Signaling Technology); COL3A1 (Cell Signaling Technology); GAPDH (CWBIO, Beijing, China).

### Hematoxylin and eosin (H&E) stain, immunohistochemistry (IHC) and immunofluorescence staining

Liver tissues from each group were fixed in 4% paraformaldehyde, embedded in paraffin, and sectioned. H&E was conducted as previously described 28. For IHC, tissue sections were deparaffinized, rehydrated, antigen-retrieved, and blocked with goat serum. Sections were incubated with primary antibodies (FAT10 and α-SMA) overnight at 4 °C. The expression of FAT10 and α-SMA was evaluated in a blind manner by two independent pathologists. Immunofluorescence staining was conducted as previously described 29.

### Masson staining

Masson staining was conducted as previously described 30.

### Statistical analyses

At least three independent experiments were performed for each experiment. Data are presented as the means ± SD and results were generated using GraphPad Prism 5.0 (San Diego, CA, USA). Two-tailed Student's *t* test was used for comparisons between two groups and one-way analysis of variance (ANOVA) was used for more than two groups. *P* < 0.05 was considered statistically significant.

## Results

### FAT10 expression is upregulated by CCl4 in HSCs

**Figure 1:**
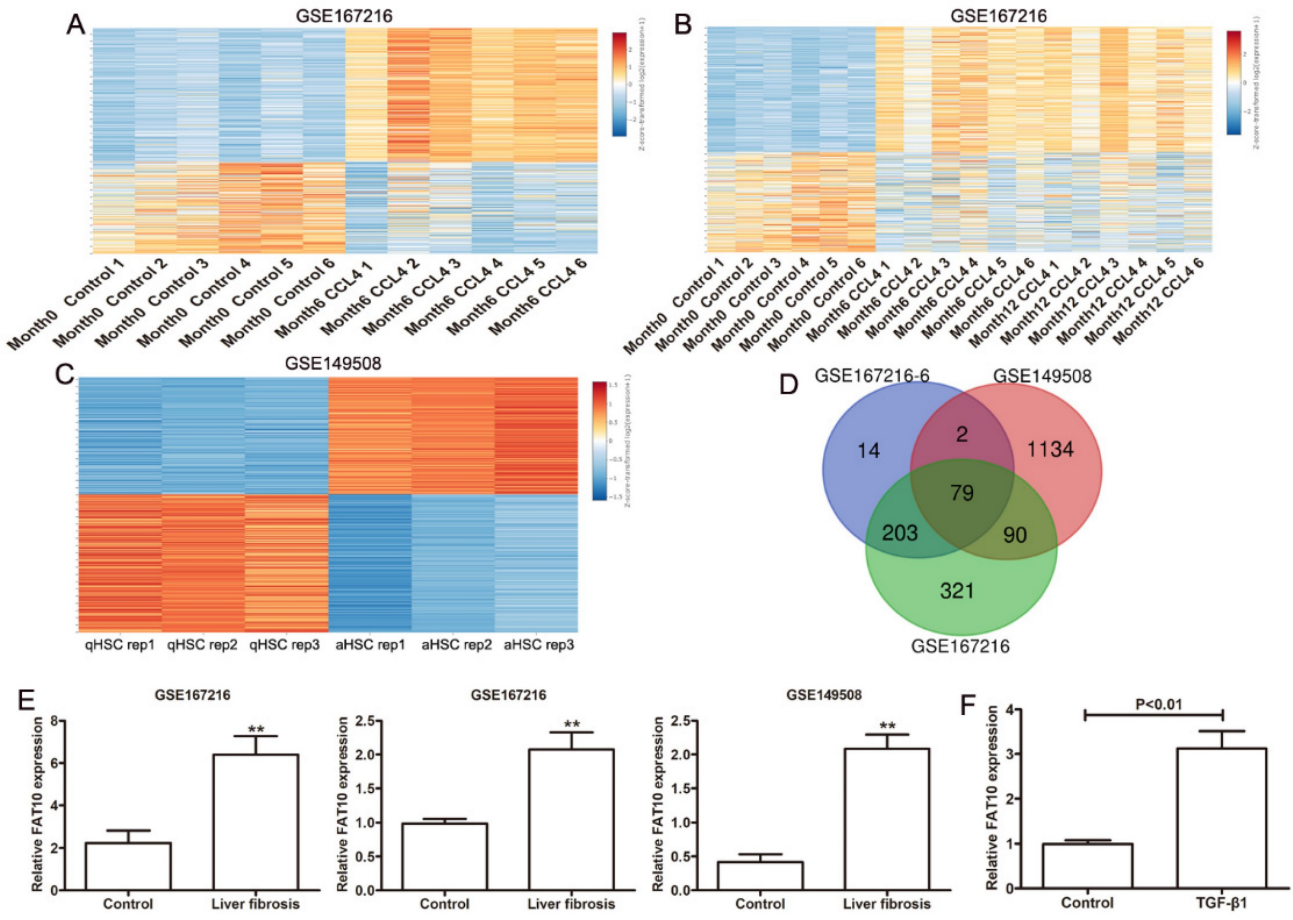
** Gene expression values among the hepatic fibrosis-related genes in liver fibrosis. (A)** Different genes in normal mouse liver and diverse liver fibrotic models (6 months CCl4-treated mice) by RNA sequencing (GSE167216) were presented in the heat map. **(B)** Heat maps showed different genes in normal mouse liver and diverse liver fibrotic models (6 and 12 months CCl4-treated mice) by RNA sequencing (GSE167216) were presented. **(C)** Different genes between quiescent and activated mouse primary hepatic stellate cell (HSC) from the GSE149508 dataset. **(D)** The Venn diagram of same genes in the above upregulated genes. **(E)** The expression of FAT10 in GSE167216 and GSE149508 dataset. **(F)** The mRNA level of FAT10 in LX-2 treated with or without TGF-β1 at 24 hours. **p < 0.01.

To detect the key genes in HSCs during the progress of liver fibrosis, GEO database (GSE167216) containing differentially expressed genes (DEGs) in C57BL/6J mice treated with or without CCl4 by high throughput sequencing were used to analyze with [|log2 fold change (FC)| > 1.5 and *P*-value < 0.01]. Using the easyGEO (https://tau.cmmt.ubc.ca/eVITTA/easyGEO_demo/), we found many genes have changed after 6 months of CCl4 administration (Figure 1A), as well as 12 months of CCl4 administration (Figure 1B). (GSE149508) containing RNA-seq data from mouse HSCs treated with or without CCl4 were used to analyzed by easyGEO, the unsupervised hierarchical clustering analysis showed the most significant DEGs between quiescent hepatic stellate cells (qHSCs) and activated hepatic stellate cells (aHSCs) with [|log2 fold change (FC)| > 1.5 and *P*-value < 0.01] (Figure 1C). Then we analyzed the all upregulated genes overlapping among liver fibrosis model by 6 months of CCl4 administration, liver fibrosis model by 12 months of CCl4 administration and HSC activation. There were 79 upregulated genes overlapping and FAT10 was chosen to the following experiments due to the roles of FAT10 in hepatocellular carcinoma (Figure 1D). The expression of FAT10 was significantly upregulated, in both activated HSCs and fibrotic liver tissues (Figure 1E). The upregulation of FAT10 was confirmed in LX-2 cells treated with TGF-β1 (Figure 1F). These findings indicated that FAT10 might play a key role in HSCs activation and liver fibrosis.

### FAT10 silencing reduced ECM activation and accumulation induced by TGF-β1 in LX-2 cells

**Figure 2:**
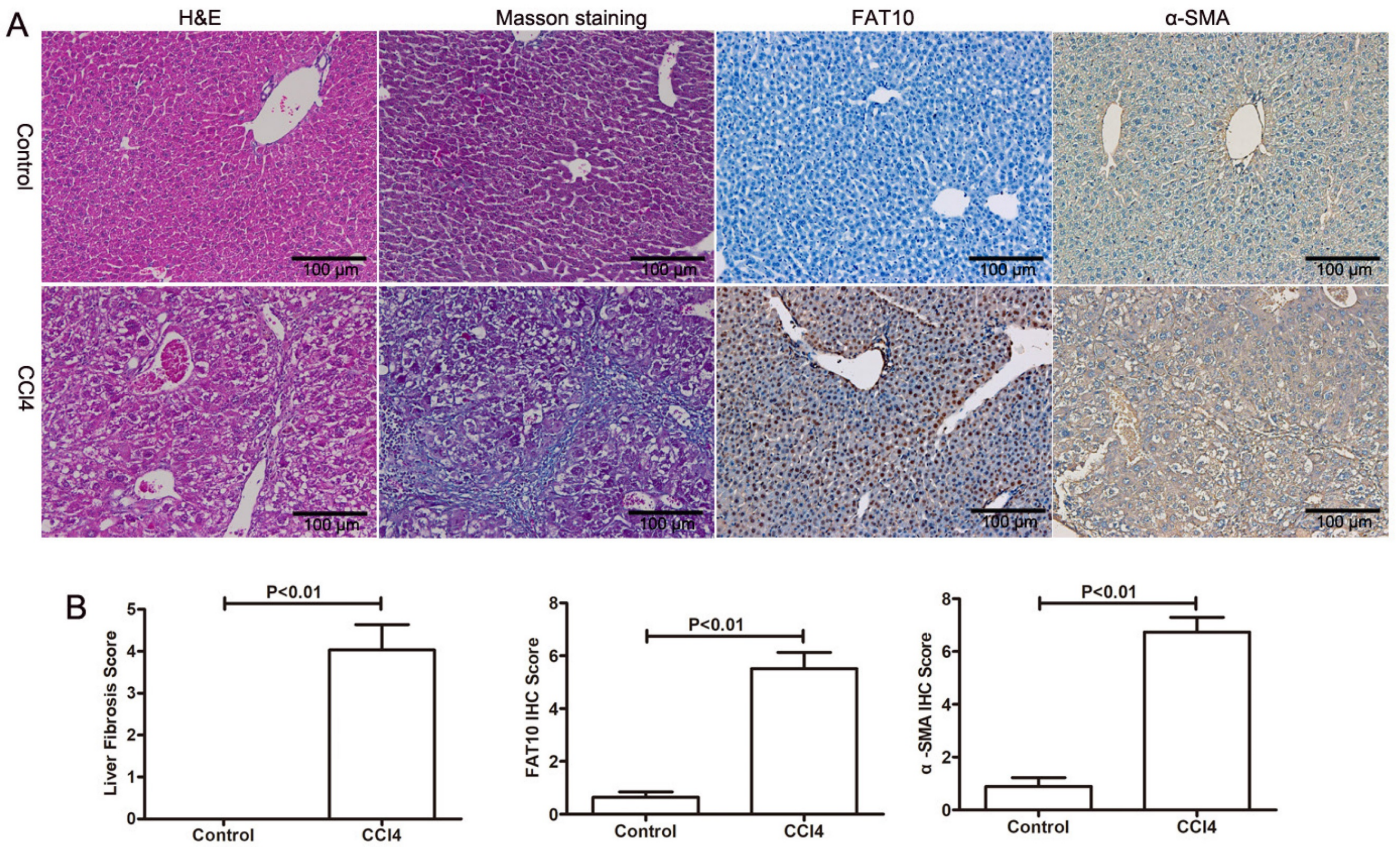
** FAT10 was upregulated in hepatic fibrosis. (A)** Mice were administered with CCl4, and liver tissues were collected for H&E, Masson's trichrome, FAT10 and α-SMA staining in each group. Magnification, 200X. **(B)** Fibrosis scores of the Masson-stained sections and the integrated optical density (IOD) of FAT10 and α-SMA staining. **p < 0.01.

To explore the role of FAT10 in HSCs activation, CCl4-induced liver injury and fibrosis were established. As shown in Figure 2A and 2B, H&E staining showed that CCl4 led to hepatocyte degeneration and inflammatory cell infiltration in liver sections, confirming the hepatic injury (Figure 2A). The results of Masson's Trichrome staining demonstrated that collagen deposition and fibrosis in liver tissues (Figure 2B). Furthermore, expression of FAT10 and α-SMA by using immune-labelling method, the expression of FAT10 was confirmed both in liver cells and HSCs of liver tissues after fibrosis and the expression of α-SMA mainly located in nucleoplasm and the expression of FAT10 was mainly located in the actin filaments.

**Figure 3:**
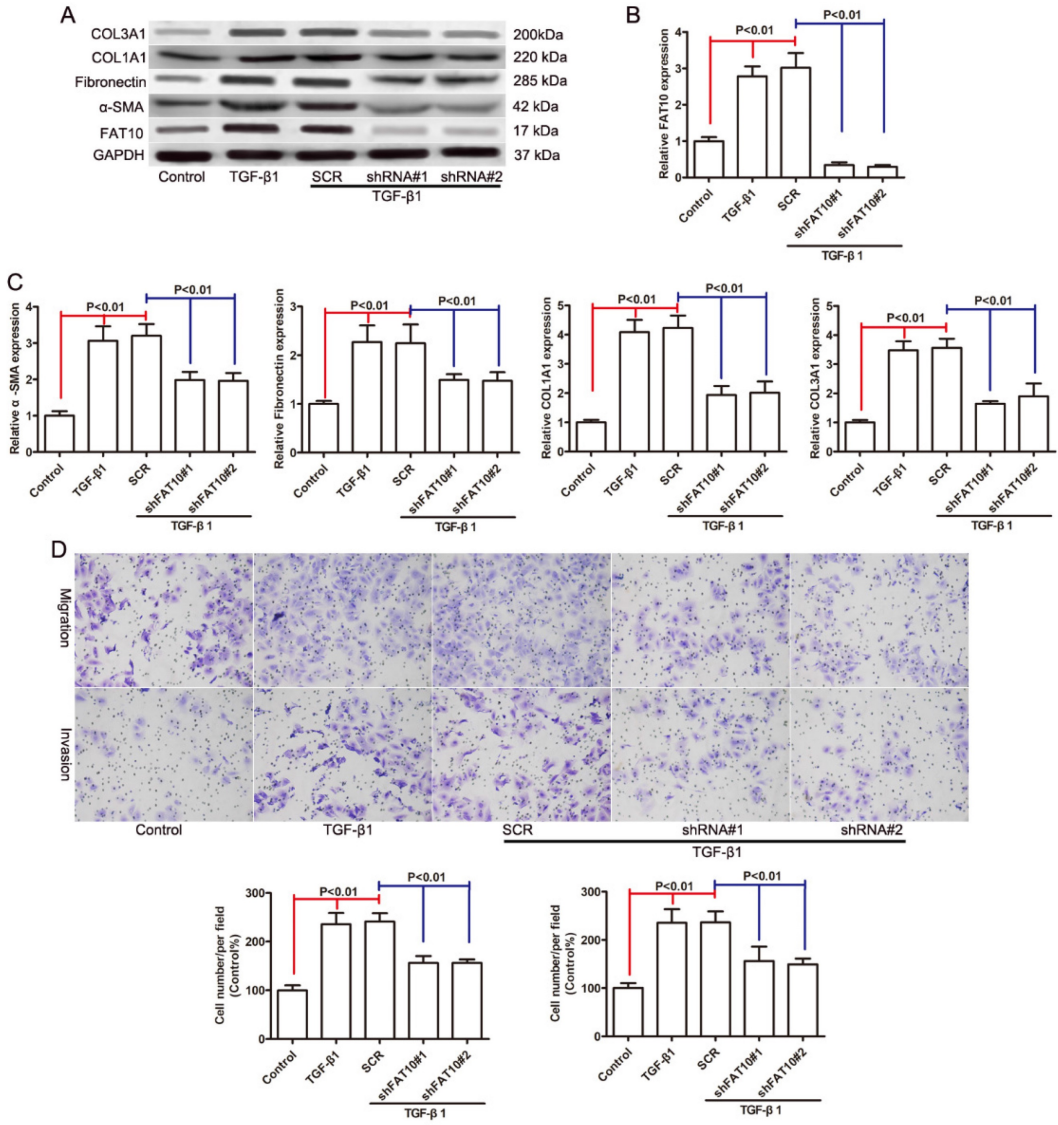
** FAT10 silencing reduced the expression of fibrosis markers in LX-2 cells activated by TGF-β1. After 24 h of TGF-β1 stimulation. (A)** The protein levels of FAT10, α-SMA, Fibronectin, COL1A1, and COL3A1 in FAT10 knockdowned LX-2 cells. **(B)** The mRNA expression of FAT10 in FAT10 knockdowned LX-2 cells. **(C)** The mRNA expression of fibrosis markers in FAT10 knockdowned LX-2 cells. (D) The representative images and quantification of the effects of FAT10 silencing on LX-2 cells migration and invasion.

Since FAT10 was significantly upregulated in HSCs activation, we used two distinct shRNAs targeting FAT10 to knock down FAT10 in LX-2 cells with SCR (a non-target shRNA) as a control to assess the biological function of FAT10 in HSCs activation. The knockdown efficiency of FAT10 was evaluated by qRT-PCR (Figure 3B) and Western blotting (Figure 3A). Then we assessed the effect of FAT10 silencing on TGF-β1 induced expression of α-SMA, Fibronectin, COL1A1, and COL3A1, which were the markers of HSCs activation. The mRNA expression levels of α-SMA, Fibronectin, COL1A1, and COL3A1 were significant increased after TGF-β1 treatment (Figure 3C), while FAT10 silencing extenuated the upregulaton of such liver fibrotic markers (Figure 3C). Consistent with the results of mRNA, immunoblotting demonstrated that protein expression was increased after TGF-β1 treatment (Figures 3A). FAT10 silencing significantly downregulated the expression of α-SMA, Fibronectin, COL1A1, and COL3A1 in LX-2 cells stimulated by TGF-β1 (Figures 3A). Further Transwell results suggested that knockdown of FAT10 could inhibit TGF-β1-induced LX-2 cell migration and invasion (Figures 3D). These data indicated that silencing of FAT10 could effectively reverse the TGF-β1-induced ECM production and HSCs activation.

### FAT10 silencing inhibited the inflammatory response in LX2 cells

**Figure 4:**
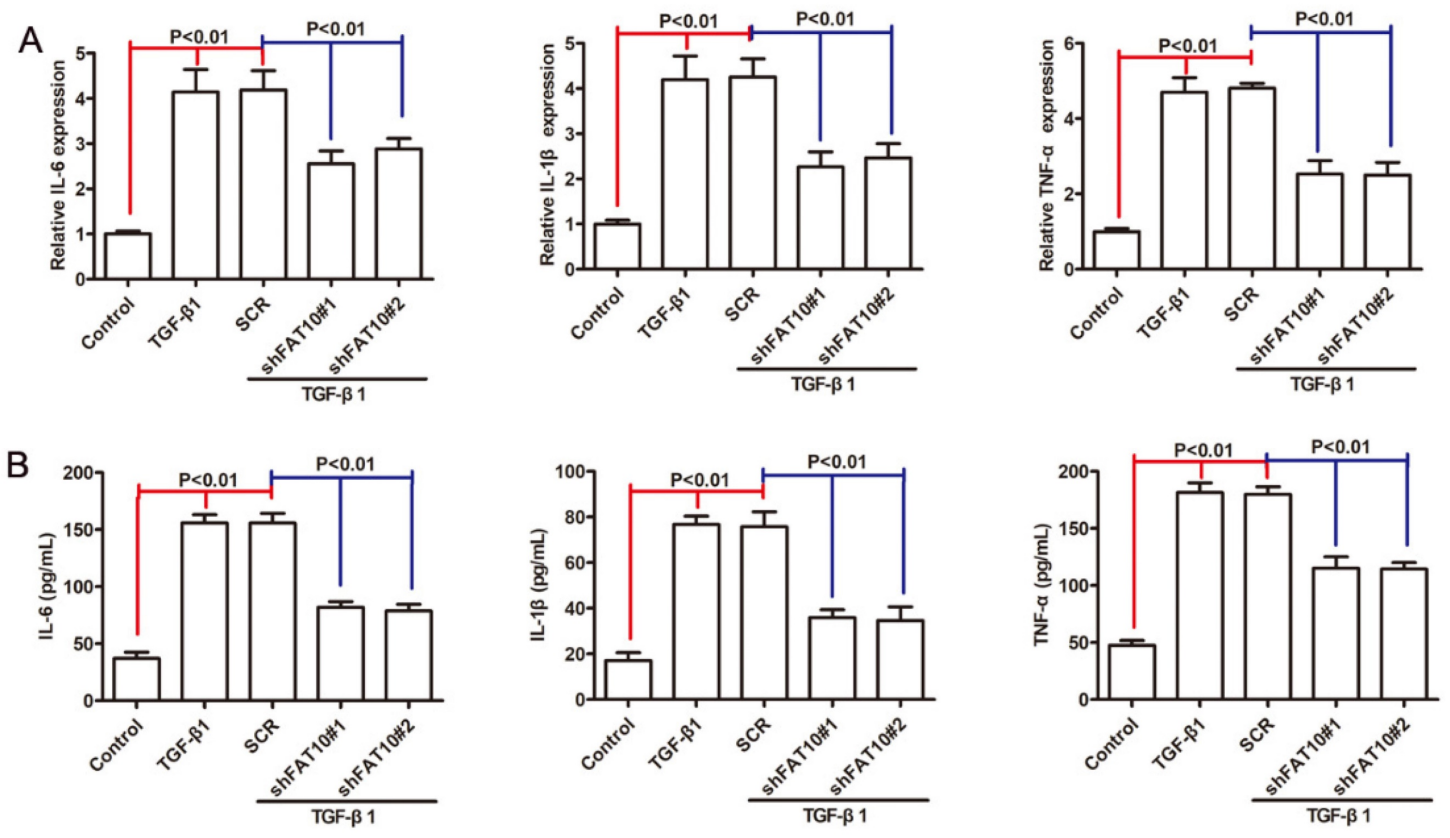
** FAT10 silencing inhibited inflammation response in TGF-β1-activated LX-2 cell. After stimulation with TGF-β1 (10 ng/ml) for 24 h in FAT10 silencing LX-2 cells. (A)** qRT-PCR analysis for inflammatory factors including IL-6, IL-1β and TNF-α. **(B)** ELISA analysis for IL-6, IL-1β and TNF-α in the collected supernatants.

Inflammation was considered as one of the crucial factors in fibrosis progression 31, 32, then we investigated the roles of FAT10 in LX-2 cells proinflammatory response after TGF-β1 treatment. As shown in Figure 4A, the mRNA levels of IL-1β, IL-6 and TNF-α were significantly increased following TGF-β1 treatment, which could be abrogated after FAT10 silencing. Similar results were observed in cellular supernatant (Figure 4B). Based on these results, we concluded that FAT10 played an important role in inflammatory activation of LX-2 cells.

### FAT10 mediated ECM accumulation and inflammatory response through SIRT1 in LX2 cells

**Figure 5:**
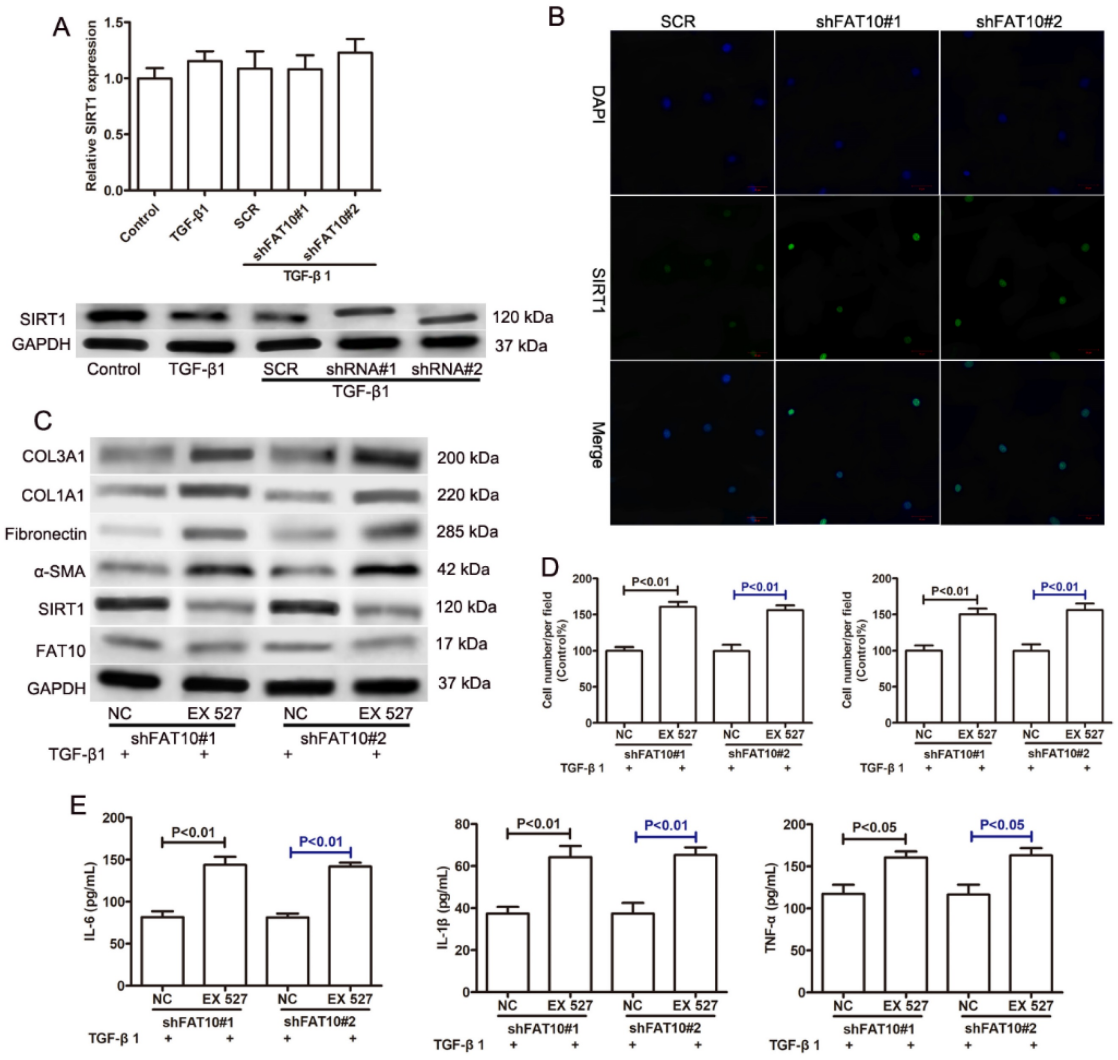
** Effects of SIRT1 on the FAT10-mediated profibrotic and inflammatory response in TGF-β1-activated LX-2 cells. (A)** The mRNA level of SIRT1 was detected using qRT-PCR as indicated treatment. The protein level of SIRT1 was detected using Western blotting as indicated treatment. FAT10 silencing LX-2 cells were pre-incubated with EX527 (SIRT1 inhibitor) for 1h, and then were incubated with TGF-β1 (10 ng/ml) for 24h. **(B)** Immunofluorescence staining of SIRT1 after FAT10 silencing, nuclei were visualized by DAPI. Magnification, 200X. **(C)** Western blotting results for the protein expression levels of FAT10, SIRT1, α-SMA, Fibronectin, COL1A1, and COL3A1 in LX-2 cells. **(D)** Quantification for LX-2 cell migration and invasion was exhibited. **(E)** IL-6, IL-1β and TNF-α contents in the collected supernatants were determined using ELISA analysis.

A previous study reported that FAT10 regulated autophagy through modulating SIRT1 degradation in ischemic myocardial injury 29. We didn't observe the differences on SIRT1 mRNA level in RNA sequencing data, which prompted us to explore the relationship between FAT10 and SIRT1 in liver fibrosis. There was no significant change on the expression of SIRT1 mRNA in LX-2 cells with or without TGF-β1 treatment. And FAT10 silencing didn't influence the expression of SIRT1 mRNA (Figure 5A). Interestingly, western blotting showed that the protein level of SIRT1 was significantly decreased in LX-2 cells after TGF-β1 treatment, but FAT10 silencing attenuated the inhibitory effect of TGF-β1 on SIRT1 protein expression (Figure 5A). Consistent with the finding, FAT10 silencing resulted in a significant increase in SIRT1, as detected by confocal microscopy as an increase in the amount of green fluorescent protein (GFP)-tagged SIRT1 (Fig. 5B). To further explore whether SIRT1 was required for FAT10-mediated ECM accumulation and inflammation, EX527, a SIRT1 inhibitor, was added in FAT10 knockdown LX-2 cells. As shown in Figure 5C, EX527 could effectively decrease the protein expression of SIRT1, but there was no significant change on the protein expression of FAT10. Importantly, the effect of reduced ECM production mediated by FAT10 silencing was completely abolished by EX527 treatment (Figure 5C). Moreover, the inhibition of cell migration and invasion caused by FAT10 silencing was reversed by EX527 treatment (Figure 5D). Furthermore, ELISA results showed that TGF-β1 stimulation could induce the upregulation of inflammatory factors in the culture supernatant of LX-2 cells, whereas this stimulating effect of TGF-β1 on the secretion of inflammatory factors could be inhibited by FAT10 silencing. Finally, EX527 could strongly abolish the aforementioned inhibitory effect of FAT10 silencing. These data suggested that TGF-β1 activated FAT10 protein expression, resulting in decreased SIRT1 protein expression, thereby stimulating ECM accumulation and inflammatory response.

## Discussion

Liver fibrosis is a common pathological condition and unavoidable pathological process caused by chronic and persistent inflammatory response 1, 3, 33. Emerging evidence has shown that activation of HSCs is a landmark event in response to injury in the progress of liver fibrosis 34, 35. In this study, our analysis of GEO datasets revealed that the mRNA level of FAT10 was significantly upregulated in fibrotic liver tissues, as well as primary HSCs isolated from mice with liver fibrosis 8, 25. Further experiments revealed that inhibiting expression of FAT10 could attenuate the ECM accumulation and inflammatory response of LX-2 under TGF-β1 stimulation by increasing the expression of SIRT1.

Many studies have shown that FAT10 predominantly acts as an oncogene in tumor progression by directly targeting its conjugation substrates for degradation by the 26S proteasome 18, 21, 24. Increased collagen-rich ECM was considered as typical characteristics of activation of HSCs 15, 35, 36. The results of our study showed that the expression of FAT10 was significantly increased in LX-2 cells after TGF-β1 stimulation, in agreement with previous RNA sequencing studies 8, 25. The *in vivo* experiments also confirmed the protein level of FAT10 was remarkably upregulated in liver tissues of CCl4-treated mice. Moreover, depletion of FAT10 could attenuate the ECM accumulation in TGF-β1-activated LX-2 cells, which indicated that FAT10 was required for activation of HSCs.

Numerous studies had shown that persistent inflammation was a pan-etiology driver of liver fibrosis 31, 37. There was evidence that FAT10 was upregulated in B cells or dendritic cells following IFN-γ and TNF-α induction under inflammatory conditions 38. The inflammatory transcription factor STAT3 collaborated with NF-κB and acted on the FAT10 promoter and then induced FAT10 gene expression, resulting in increased FAT10 expression in inflammation-related cancer types 22, 24, 38. Similarly, in our present study, we found that higher levels of inflammatory factors were observed in TGF-β1-stimulated LX-2 cells compared to controls, and inhibition of FAT10 almost abrogated the upregulation of inflammatory factors, which indicated the elevated inflammatory response might be mainly attributed to up-regulation of FAT10 gene expression.

SIRT1, a highly conserved NAD+-dependent deacetylase, played an important role in anti-fibronectin and anti-inflammatory properties 39, 40. Recent study had shown that FAT10 modulated SIRT1 degradation via its C-terminal glycine residues to inhibit SIRT1 nuclear translocation and reduce SIRT1 activity 29. Consistent with the results, no significant changes in SIRT1 mRNA levels were observed following FAT10 silencing in LX-2 cells as well as in the liver tissue of CCl4-treated mice. However, the protein level of SIRT1 was remarkably increased after FAT10 silencing, accompanied with the downregulation of α-SMA, Fibronectin, COL1A1, and COL3A1, which indicated that SIRT1 was regulated by FAT10 at a post-translational regulation. The results appeared to be in accord with the essential roles of SIRT1 in ameliorating liver fibrosis 41, 42. In addition, the SIRT1 inhibitor EX527 reversed the inhibition effects on ECM accumulation and inflammatory response in LX-2 cells after FAT10 silencing, suggesting that SIRT1 protein was involved in FAT10-mediated liver fibrosis. There were some limitations in our research, such as the proteasome system and the roles of FAT10 *in vivo*. More studies are needed to investigate the precise molecular mechanisms.

## Conclusion

The present study demonstrated that FAT10 could down-regulate the protein expression of SIRT10 at the post-translational level, thereby activating hepatic stellate cells and leading to liver fibrosis. Targeting FAT10 may represent a new therapeutic strategy for the treatment of liver fibrosis.

## Figures and Tables

**Table 1:** List of the primers

GAPDH	Forward Primer	ACAACTTTGGTATCGTGGAAGG
	Reverse Primer	GCCATCACGCCACAGTTTC
FAT10	Forward Primer	CCGTTCCGAGGAATGGGATTT
	Reverse Primer	GCCATAAGATGAGAGGCTTCTCC
Fibronectin 1	Forward Primer	AGGAAGCCGAGGTTTTAACTG
	Reverse Primer	AGGACGCTCATAAGTGTCACC
COL1A1	Forward Primer	ATCAACCGGAGGAATTTCCGT
	Reverse Primer	CACCAGGACGACCAGGTTTTC
α-SMA	Forward Primer	GTGTTGCCCCTGAAGAGCAT
	Reverse Primer	GCTGGGACATTGAAAGTCTCA
COL3A1	Forward Primer	GGAGCTGGCTACTTCTCGC
	Reverse Primer	GGGAACATCCTCCTTCAACAG
IL-6	Forward Primer	ACTCACCTCTTCAGAACGAATTG
	Reverse Primer	CCATCTTTGGAAGGTTCAGGTTG
IL-1β	Forward Primer	AGCTACGAATCTCCGACCAC
	Reverse Primer	CGTTATCCCATGTGTCGAAGAA
TNF-α	Forward Primer	CCTCTCTCTAATCAGCCCTCTG
	Reverse Primer	GAGGACCTGGGAGTAGATGAG

## Abbreviations

FAT10: human leukocyte antigen (HLA)-F adjacent transcript 10
SIRT1: sirtuin 1
ECM: extracellular matrix
HSCs: hepatic stellate cells
GEO: Gene Expression Omnibus
CCl4: Carbon tetrachloride
α-SMA: alpha-smooth muscle actin
HCC: hepatocellular carcinoma
DEGs: differentially expressed genes
qRT-PCR: Quantitative Real-Time polymerase chain reaction
shRNA: short hairpin RNA
ELISA: Enzyme-Linked Immunosorbent Assay
H&E: Hematoxylin and eosin
IHC: Immunohistochemistry
TGF-β1: transforming growth factor beta 1
NAD+: nicotinamide adenine dinucleotide
